# Gene expression deregulation by *KRAS *G12D and G12V in a *BRAF *V600E context

**DOI:** 10.1186/1476-4598-7-92

**Published:** 2008-12-16

**Authors:** Massimiliano Monticone, Emanuela Biollo, Massimo Maffei, Alessandra Donadini, Francesco Romeo, Clelia Tiziana Storlazzi, Walter Giaretti, Patrizio Castagnola

**Affiliations:** 1 Centro Biotecnologie Avanzate, Genova, Italy; 2 Dipartimento di Chimica e Tecnologie Farmaceutiche ed Alimentare, Università di Genova, Genova, Italy; 3 Istituto Nazionale per la Ricerca sul Cancro, Genova, Italy; 4 Dipartimento di Genetica e Microbiologia, Università di Bari, Bari, Italy

## Abstract

**Background:**

*KRAS *and *BRAF *mutations appear of relevance in the genesis and progression of several solid tumor types but the co-occurrence and interaction of these mutations have not yet been fully elucidated. Using a microsatellite stable (MSS) colorectal cancer (CRC) cell line (Colo741) having mutated *BRAF *and *KRAS*^*WT*^, we also aimed to investigate the *KRAS-BRAF *interaction. Gene expression profiles for control *KRAS*^*WT*^, *KRAS*^*G*12*V *^and *KRAS*^*G*12*D *^transfected cells were obtained after cell clone selection and RT-PCR screening. Extensive qPCR was performed to confirm microarray data.

**Results:**

We found that the *KRAS*^*G*12*V *^state deregulated several genes associated to cell cycle, apoptosis and nitrogen metabolism. These findings indicated a reduced survival and proliferation with respect to the *KRAS*^*WT *^state. The *KRAS*^*G*12*D *^state was, instead, characterized by several other distinct functional changes as for example those related to chromatin organization and cell-cell adhesion without affecting apoptosis related genes.

**Conclusion:**

These data predict that the G12D mutation may be more likely selected in a *BRAF *mutated context. At the same time, the presence of the *KRAS*^*G*12*V *^mutation in the cells escaping apoptosis and inducing angiogenesis via IL8 may confer a more aggressive phenotype. The present results get along with the observations that CRCs with G12V are associated with a worse prognosis with respect to the WT and G12D states and may help identifying novel CRC pathways and biomarkers of clinical relevance.

## Background

Normal colon epithelial cells, in their way to malignancy, may follow multiple pathways: i) the traditional adenoma-carcinoma sequence associated with chromosomal instability, in which the sequential accumulation of mutations in specific genes, including adenomatous polyposis coli (*APC*), v-Ki-ras2 Kirsten rat sarcoma viral oncogene homolog (*KRAS*), and tumor protein p53 (*TP53*), drives the transition from healthy colonic epithelia through increasingly dysplastic adenoma to colorectal cancer (CRC) [1] ii) the serrated pathway leading to CRC associated with microsatellite instability (MSI), v-raf murine sarcoma viral oncogene homolog B1 (*BRAF*) mutations and extensive DNA methylation [2,3] and possibly, iii) a "fusion" pathway associated to methylation of the O-6-methylguanine DNA methyltransferase (*MGMT*), mutation of *KRAS *and inactivation of the gene coding for tumor protein p53 (*TP53*) [4].

*KRAS *is one of the most commonly activated oncogenes since 17% to 25% of all human tumors harbor mutations in this gene [5]. Although statistics may differ slightly from study to study, a good estimate is that in about 30–40% of CRC a mutated *KRAS *may be found [6-9]. Ras proteins are small guanine-nucleotide binding proteins (p21ras) involved in signal transduction with a GTPase activity, which is severely reduced when the protein is mutated in codons 12, 13 or 61. As p21ras activates downstream effectors in the GTP-bound state, reduction of this activity leads to unregulated signaling and lastly to enhanced and unregulated cell proliferation and transformation [10].

Of the multiple molecular signaling pathways initiating from *KRAS*, the Raf/MEK/ERK kinases and the Ras/PI3K/PTEN/Akt pathways are the best studied [11,12]. These pathways are interconnected since the mutation of genes in one pathway may influence the activity of kinases in the other pathway and both of them also interact with the *TP53 *pathway [12]. Because of these molecular interactions, the effects of the activation of one of these pathways may be very different in different cellular context [13,14] and may result in complex functional effects including changes in cellular proliferation, cell cycle, chromosomal instability, apoptosis, drug resistance and prognosis [8,12,15-17]. Also the role of the KRAS-BRAF interaction (being BRAF an effector of RAS in the RAF and PI3K activated pathways) is far from being understood.

Although *BRAF *mutations have been observed mainly in sporadic MSI CRC, they have also been detected in a small percentage of microsatellite stable (MSS) CRCs [18-22]. In particular, *BRAF *mutations were more often found in premalignant colon polyps and in early, rather than in advanced, CRC [18,23-25]. As concomitant *KRAS *and *BRAF *mutations are quite rare in premalignant colon polyps and early stages of CRC, they are considered as alternative or mutually exclusive mutations [26,27]. In a recent study, however, it was found that the number of concomitant *KRAS *and *BRAF *mutations increased along with the depth of the wall invasion of sporadic MSS CRC, suggesting that activation of both genes is likely to harbor a synergistic effect and that *KRAS *could give the tumor an invasive behavior [28]. Therefore, cells harboring a *BRAF *mutation would represent a model system with a genetic background well suited to study the specific contribution of activating *KRAS *mutations to CRC progression. To this end, we have used the colorectal adenocarcinoma cell line Colo741, which is wild-type relatively to *KRAS*, MSS [29] but harbors a mutation (V600E; single letter amino acid code) in the *BRAF *locus [30], and stably transfected these cells with constructs expressing the *KRAS*^*G*12*V *^or the *KRAS*^*G*12*D *^mutated coding sequences (cds) or the *KRAS*^*WT*^. We selected these *KRAS *mutations since codon 12 is the most affected by point mutations in CRC (more than 90%) and because, among all mutation types in sporadic MSS CRC, G12D and G12V, they are the most frequently observed with a frequency of about 45% and 23% respectively [9].

## Results

### Mutated KRAS expression modifies the gene expression profile of Colo741 cells

**Figure 1:**
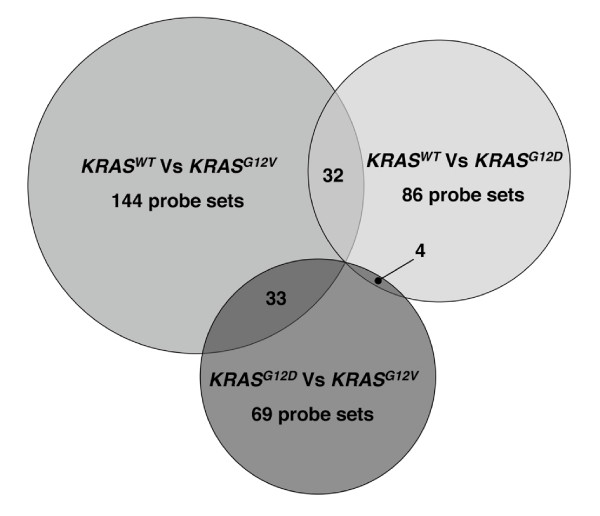
Venn diagrams obtained by SAM analysis. Global comparison among the genes regulated by the *KRAS*^*G*12*D*^, *KRAS*^*G*12*V *^and *KRAS*^*WT *^isoforms expressed by transfection in Colo741 cells. The number of probe sets associated to the co-regulated genes is reported in the overlapping areas.

The effects of mutated *KRAS *on gene expression were investigated by performing GeneChip microarray studies. Data from probed and scanned arrays (two technical replicates were analyzed for the three conditions: *KRAS*^*WT*^, *KRAS*^*G*12*D *^and *KRAS*^*G*12*V*^) were normalized, filtered by removing probe sets that were regarded as not expressed and then analyzed by performing a multi-class of all 6 arrays using the SAM program. A change in gene expression was considered significant if the *p *value was less than 0.02 and increased gene expression occurred in, at least, one out of the three conditions. We also performed a two-class unpaired comparison for *KRAS*^*G*12*D *^*versus KRAS*^*WT *^and *KRAS*^*G*12*V*^*versus KRAS*^*WT *^to specify expression changes. We chose a 2.0 fold change cutoff. Based on these criteria, 25 probe sets were up regulated and 61 down regulated in *KRAS*^*G*12*D*^*versus KRAS*^*WT*^. In the comparison *KRAS*^*G*12*V*^*versus KRAS*^*WT*^, 88 probe sets resulted up regulated and 56 down regulated (see Additional File 1). Then, another two-class unpaired comparison between the mutated *KRAS*-expressing Colo741 found 52 and 17 probe sets respectively up- or down-regulated (see Additional File 2). In total, irrespective of the comparison, we found 229 regulated probe sets (Figure 1) corresponded to 219 unique genes.

### Analysis of gene expression profiles

**Figure 2:**
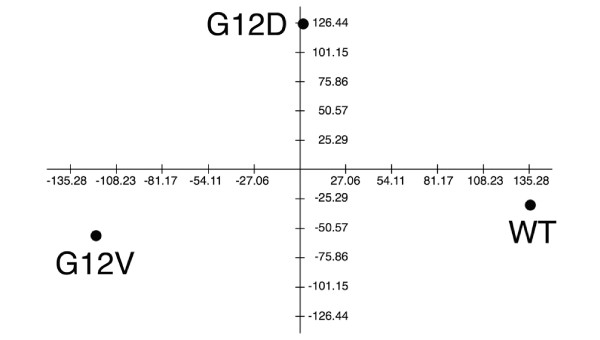
Microarray analysis performed with TIGR MeV program: principal component analysis. Microarray analysis of Colo741 cell clones transfected with constructs expressing the *KRAS*^*G*12*D *^(G12D), *KRAS*^*G*12*V *^(G12V) and *KRAS*^*WT *^(WT) isoforms. Probe sets associated to dysregulation of gene expression levels among the three groups were identified using SAM (see Materials and Methods). The corresponding values from two independent microarray analysis were averaged. Principal component analysis (PCA) is shown to provide the 2D projections onto the plane spanned by the two principal components for the three different *KRAS *profiling data sets.

All the differentially 229 expressed probe sets, after creating a single sample by averaging the individual expression values from technical replicas, were then analyzed with the software tool TIGR MeV. Analysis of mutated *KRAS*-expressing Colo741 clones by PCA revealed a distinct partition among those expressing the recombinant *KRAS*^*WT *^and those expressing the *KRAS*^*G*12*D *^and *KRAS*^*G*12*V *^(Figure 2). Points in the two-dimensional plot represented the samples. The distance between any pair of points was related to the similarity between the two observations in high-dimensional space. Samples that were near each other in the plot were similar in a large number of variables, i.e., expression level of individual genes. Conversely, samples that were far apart in the plot were different in a large number of variables.

**Figure 3:**
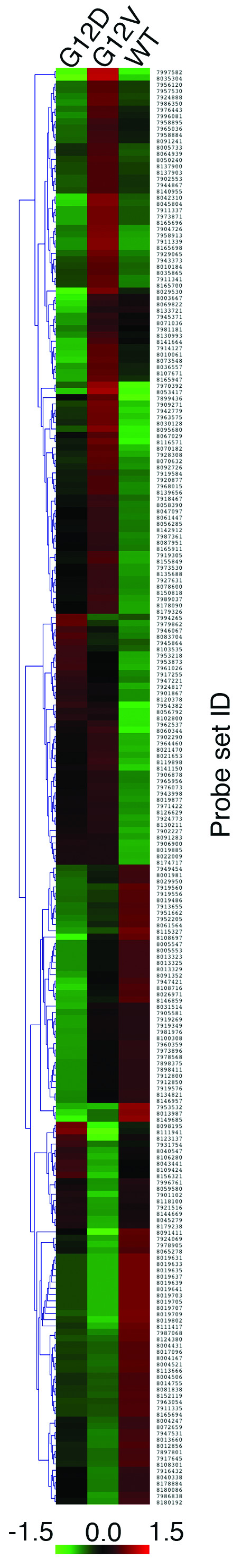
Microarray analysis performed with TIGR MeV program: hierarchical clustering. Heat map visualization obtained by hierarchical clustering (HCL). Ratios for each probe relative to the mean value (calculated from the two independent microarray analysis for each condition) were used to rearrange the gene list on the basis of their expression pattern. Probes corresponding to genes with similar regulation trend were placed close to each other. The color-ratio bar indicates intensity of gene up-regulation (red), down-regulation (green) and no change (black).

Differential expression among the three *KRAS *conditions was visualized by hierarchical clustering (HCL) which generates a tree (dendogram) to group similar objects together (Figure 3).

### Expression patterns and biological pathways specifically identified by EASE in KRAS-expressing clones

To gain a more mechanistic understanding of the main processes affected by the *KRAS*, the EASE score [31] was used to identify Gene Ontology (GO) functional categories, which were significantly over-represented (see Additional Files 1 and 2). After filtering the results to avoid redundant and/or generic categories, statistically significant GO terms associated with *KRAS*-regulated genes were found (Table 1). This analysis identified cell cycle arrest and apoptosis genes as being the most affected ones by *KRAS*^*G*12*V*^. When genes regulated by *KRAS*^*G*12*D *^(see Additional File 1) were subjected to EASE analysis, genes involved in cellular component organization and biogenesis were identified. Finally, we chose to examine the 69 probe sets differently regulated between mutated *KRAS*-expressing Colo741 (see Additional File 2) in order to compare the specific effects of the two oncogenes *KRAS*^*G*12*V *^and *KRAS*^*G*12*D*^. Again, we used the EASE score to perform ontological categorization and KEGG pathway analysis. These analyses identified genes associated to immune system processes and to the biosynthesis of steroids as the most affected ones (Table 1).

**Table 1:** Gene Ontology analysis and KEGG pathway analysis of KRAS isoform-expressing Colo741 cell clones.

**System Gene Category – Term**	**Count**	**%**	**P-Value**	**Genes**
***KRAS*^*G*12*V *^*Vs KRAS*^*WT*^**				
**GO biological process**				
Cell cycle arrest	6	6.7	5.50E-05	*DDIT3, DHCR24, GADD45A, IL8, PPP1R15A, SESN2*
Apoptosis	13	14.6	3.90E-04	*ANXA1, APOE, GADD45A, IFIH1, IL24, DDIT3, DDIT4, DHCR24, PMAIP1, PPP1R15A, SEMA6A, TNFRSF19, TRIB3*
**KEGG pathway**				
Nitrogen metabolism	3	3.4	1.50E-02	*ASNS, CTH, GLS*

***KRAS*^*G*12*D *^*Vs KRAS*^*WT*^**				
**GO biological process**				
Cellular component organization and biogenesis	12	27.3	5.10E-02	*CRYAB, EHD2, FHOD1, HIST1H1A, HSPB1, LIN7C, MAP3K11, PCDHB5, PCDHB16, SEMA6A, SLC7A11, SMCHD1*

***KRAS*^*G*12*D *^*Vs KRAS*^*G*12*V*^**				
**GO biological process**				
Immune system process	14	32.6	3.50E-07	*BST2, CDK6, IFITM3, IFIT1, IFI27, IFI44, IL8, IL24, MICA, OAS1, OAS2, OAS3, SYK, S100B*
Sterol metabolic process	6	14	1.10E-06	*APOE, FDFT1, HMGCR, HMGCS1, IDI1, SC4MOL*
**KEGG pathway**				
Biosynthesis of steroids	4	9.2	5.70E-05	*FDFT1, IDI1, HMGCR, SC4MOL*

Gene name symbols used are those approved by the Human Genome Organisation Gene Nomenclature Committee .

Non-redundant functional categories, number of genes contained within each category, percentages ranked by the degree of over-representation in the category as determined by EASE (P-value) and gene members found to be modulated by the *KRAS *isoforms are shown. Redundant categories with similar gene members were removed to yield a single representative category.

### Quantitative RT-PCR validated the Microarray data

**Figure 4:**
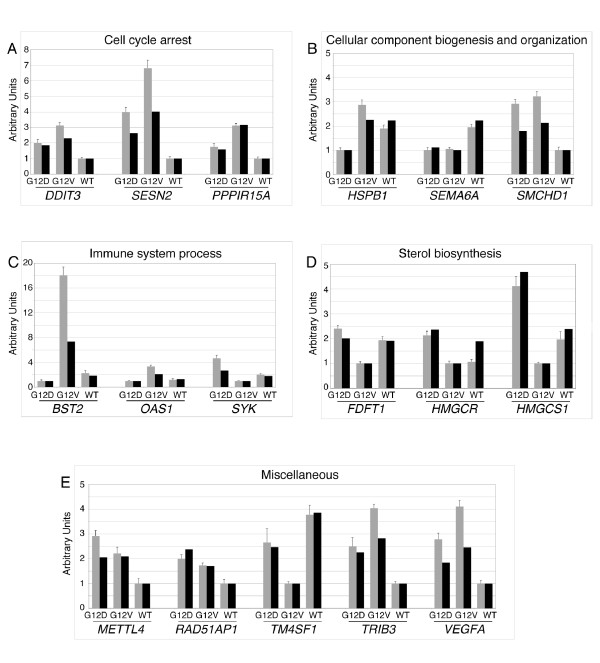
**Real-Time RT-PCR validation of microarray data**. Real-Time RT-PCR analysis performed on Colo741 cell clones transfected with constructs expressing the KRAS^G12D ^(G12D), KRAS^G12V ^(G12V) and KRAS^WT ^(WT) isoforms to validate the microarray data. This was accomplished on randomly selected genes from Table 1 and showed, in arbitrary units, KRAS isoform-dependent regulation of cell cycle arrest genes (A), of cellular component organization and biogenesis genes (B), of immune system process genes (C) or sterol metabolic process genes (D). Other *KRAS *isoform-regulated genes associated to miscellaneous functions and randomly selected from Tables S1 and S2, are shown in (E). Real-Time RT-PCR and microarray data are respectively indicated by gray and black bars. Expression levels are relative to the expression of the housekeeping Ribosomal protein L19 gene (*RPL19*). Standard deviations of Real-Time RT-PCR data are indicated as vertical bars. Gene name symbols used are those approved by the Human Genome Organisation Gene Nomenclature Committee .

To verify and validate the GeneChip microarray data, we performed real-time RT-PCR (Figure 4) on a subset of seventeen *KRAS*-modulated genes. RNA samples subjected to RT-PCR were identical to those used for the microarray analysis. In particular, we confirmed the regulated expression patterns of genes chosen on the basis of their association with cell cycle arrest processes [DNA-damage-inducible transcript 3 (*DDIT3*), sestrin 2 (*SESN2*), and protein phosphatase 1, regulatory (inhibitor) subunit 15A (*PPP1R15A*)], cellular component organization and biogenesis [heat shock 27 kDa protein 1 (*HSPB1*), semaphorin 6A (*SEMA6A*) and structural maintenance of chromosomes flexible hinge domain containing 1 (*SMCHD1*)], immune system processes [bone marrow stromal cell antigen 2 (BST2), 2',5'-oligoadenylate synthetase 1 (*OAS1*) and spleen tyrosine kinase (*SYK*)], as well as genes known to be regulated during sterol biosynthesis [farnesyl-diphosphate farnesyltransferase (*FDFT1*), 3-hydroxy-3-methylglutaryl-Coenzyme A reductase (*HMGCR*) and 3-hydroxy-3-methylglutaryl-Coenzyme A synthase 1 (*HMGCS1*)]. All validation results, on changes in mRNA expression of these genes, were proved to be regulated coherently with the GeneChip microarray data (Figure 4). In summary, the two methodologies (real time PCR and microarray) produced highly consistent results, which provided a good level of assurance regarding the validity of the microarray data.

## Discussion

The purpose of this study was to investigate by a genome wide transcription profiling approach the specific gene expression modulation due to the two most frequently occurring *KRAS *mutations in CRC (respectively, G12V and G12D) in *BRAF *mutated CRC-derived cells. The gene expression profiles associated to these mutations transfected in the host CRC Colo741 cells, characterized by mutated *BRAF *and wild type *KRAS *and evaluated versus the transfected WT isoform, were not overlapping and could be clear-cut discriminated. Furthermore, the Principal Component Analysis performed in this study made it evident that the highest degree of change in gene expression was to be associated to the G12V state.

Both G12D and G12V states appeared to co-regulate genes associated to biological processes which are highly correlated to cancer. The expression of *KRAS*^*G*12*V*^, in particular, modulated a series of genes involved in cell cycle control, apoptosis and nitrogen metabolism so that these cells are likely to undergo cell death and/or lower proliferation with respect to the cells expressing the *KRAS*^*WT *^isoform. Thus, it appears at first sight that the expression of *KRAS*^*G*12*V *^might explain why, at least at the early stages of CRC genesis, concomitant *KRAS *and *BRAF *mutations are rarely occurring. However, *BRAF*^*V*600*E *^was shown to induce genomic instability promoting the acquisition of additional genetic defects [32] and, in the specific context of MSS CRC, concomitant *BRAF *and *KRAS *mutation occurrence was shown to increase with colon wall invasion and metastases [28]. Interestingly, Costa and coworkers reported very recently a strong link between tumor recurrence, distant metastases, survival and *BRAF*^*V*600*E *^plus *RAS *mutation in thyroid carcinoma [33]. It is tempting to suggest that specific *KRAS *and *BRAF *mutation interaction may have a role to modulate gene expression profiling in specific tumor types (either MSS, or MSI, or CIN) toward a more aggressive phenotype. In our view, it is not a single gene or a given genetic system that may control tumor progression. In fact, the gene expression patterns appear modulated by the genome context coupled with the effects of different gene mutations [34].

Our data also showed that IL8 was upregulated by *KRAS*^*G*12*V*^. With this in mind, we considered of high interest previous reports showing that *HRAS*^*G*12*V*^-induced IL8 expression plays a critical role in tumor growth and angiogenesis [35], that the degree of its expression was associated with the CRC induction and progression including the development of liver metastases [36,37], and that IL8 was a central element in CRC-specific gene network [38]. We are therefore tempted to speculate that the presence of *KRAS*^*G*12*V *^in those cells which might escape apoptosis may confer an aggressive phenotype by inducing angiogenesis via IL8 and possibly facilitate metastasis.

Our results also showed that the genes regulated by the *KRAS*^*G*12*D*^isoform were related to the cellular component organization and biogenesis but not to apoptosis nor cell stress but instead it downregulated indeed at least two genes coding for chaperone proteins (*HSPB1 *and *CRYAB*). Since we showed that the G12V mutation generated more stressful conditions favoring cell cycle arrest and apoptosis than the G12D, it appears more likely that the BRAF mutation is associated with G12D. Interestingly, this observation appears in agreement with the data reported by Costa and collaborators in thyroid carcinoma [33] and by Oliveira and coworkers investigating the CRC [28].

It is known that both *KRAS *mutations under study greatly reduce the *KRAS *GTPase activity, locking the protein in a constitutively active state [39] To our knowledge, however, the crystal structures of these two *KRAS *isoforms have not yet been determined and compared. Therefore, possible different protein-protein interactions and affinities of interactions with downstream *KRAS *effectors cannot be ruled out. Interestingly, these mutations were shown to affect differently the structural conformation of the highly related HRAS protein, suggesting that differences between the switch I region of G12D and G12V Ras could modify interactions with downstream effectors [39].

The present sets of genes modulated by the two *KRAS *mutations investigated were quite different (as analyzed in details in the following) and may be partly explaining the differences observed in other studies addressing their correlation with in vitro invasion properties [40], survival of CRC patients [8] and chromosomal instability and aneuploidy [41].

Among the present downregulated genes in the two *KRAS *mutations with respect to the WT isoform, we observed *SPARC, TRPM1, SEMA6A *and *ENO2*. Reduced levels of the gene coding for *SPARC *was associated with therapy-refractory CRC [42] and inactivation of *SPARC *was related to rapid progression of CRC [43]; *TRPM1 *expression was found to decline with an increased degree of aggressiveness of the melanoma [44]; downregulation of *SEMA6A *was observed in ovarian carcinoma cell lines resistant to several chemotherapeutic drugs [45] and its extracellular domain was shown to be able to inhibit angiogenesis [46]. Concerning *ENO2*, literature reports indicated upregulation in cancer [47,48] rather than downregulation, as it occurred in our mutant transfected cells, but additional studies are needed to find any specific correlation of this subunit of the enolase in CRC and *KRAS*/*BRAF *mutations.

For the other genes that presently resulted co-regulated by the two mutated *KRAS *isoforms with respect to the WT isoform, we could not find in the literature a correlation with cancer. Consequently, we suggest that these genes deserve a future careful investigation as they might represent possible novel CRC markers.

Among the genes upregulated by the *KRAS*^*G*12*V*^, we observed *DDIT3, PPP1R15A, SESN2, APOE, DDIT3, DDIT4 ASNS*, and *CTH*. All these genes are known to be induced by a variety of stressors (including unfolded protein, endoplasmic reticulum stress, DNA damage, oxidative stress, amino acid deprivation, acidosis) [49-58] and since *IL24 *which was also upregulated in our study, is able to activate the unfolded protein response [59] and cell cycle arrest [60], it is very likely that the expression of *KRAS*^*G*12*V *^in cells already expressing a mutated *BRAF *unleashes a cascade of events leading to cell stress and hence possibly to apoptosis and inhibition of cell cycle progression. It is interesting to note that it was recently demonstrated that *HRAS*^*G*12*V *^but not *BRAF *V600E engages a rapid cell-cycle arrest mediated by the endoplasmic stress response in melanocytes [61]. The upregulation of transcription of genes related to stress response resulting from our experiment is in agreement with previous reports, albeit obtained with fibroblasts expressing *HRAS*^*G*12*V*^, suggesting that Ras is part of the stress sensing machinery [62]. Furthermore, the downregulation in cells transfected with *KRAS*^*G*12*V *^of genes involved in sterol metabolic processes appeared to us worth to note. Interestingly, there is a large number of published studies showing that products of the mevalonate pathway are essential to the post-translational processing and function of nuclear lamins, small G proteins (including Ras), and growth factor receptors constituting a survival pathway that when inhibited induces apoptosis and inhibits angiogenesis [63-65]. The downregulation of the *DHCR24 *in the cells transfected with the *KRAS*^*G*12*V *^isoform with respect to the WT isoform goes in the same line since this gene was reported to be associated to resistance to oxidative stress-induced apoptosis [66]. On the other hand, the upregulation of *TNFRSF19 *may suggest that a caspase independent cell death may take place in these cells, as already shown for 293T cells [67] and that this same gene might promote cell growth as reported for melanoma cells [68]. The significance of an upregulation of genes implicated in the immune processes in our Colo741 expressing the *KRAS*^*G*12*V *^with respect to *KRAS*^*G*12*D *^was less clear.

Among the genes associated with the *KRAS*^*G*12*D *^we observed two genes, *HIST1H1A *[69] and *SMCHD1*[70], related to chromatin organization; *PCDHB5 *and *PCDHB16 *[71] and *LIN7C *[72] implicated in cell-cell adhesion; *CRYAB *[73] and *FHOD1 *[74] in cytoskeleton organization; *EHD2 *[75] in receptor internalization; *SLC7A11 *[76] in amino acid transport; *HSPB1 *[77,78] in cellular stress response; *MAP3K11 *[79] in invasive activity; *SEMA6A *[80] in axon guidance and retrograde signaling. Given the variety of processes that may be affected by the *KRAS*^*G*12*D *^isoform gene expression modulation, the interpretation of our data appears quite complex. Nevertheless, since reduction of *MAP3K11 *and increase of *LIN7C *have been shown to facilitate respectively the in vitro invasive activity [79] and the oncogenic activation of PI3K [72], we suggest that the concordant modulation of these genes by the *KRAS*^*G*12*D *^isoform in Colo741 cells may be functional to an higher aggressive phenotype with respect to the sole presence of the *BRAF *mutation. The downregulation of *SEMA6A *points to the same direction (see also above). Moreover, the downregulation of *EHD2 *may lead to increasing the growth signaling at the cell membrane by reduction of the internalization of growth receptors. Similarly, the downregulation of the proteins involved in cell-cell adhesion (*PCDHB5 *and *PCDHB16*) could possibly promote cell delamination and cell migration.

A further comment on our present expression data is that Colo741 cells expressing the *KRAS*^*G*12*D *^isoform were not apoptosis-prone or stressed cells since they did not upregulate genes induced by cellular stressors with respect to cells expressing the *KRAS *WT isoform. Conversely, they downregulated indeed at least two genes coding for chaperone proteins (*HSPB1 *and *CRYAB*).

In relationship with all these observations and list of genes that we have specifically considered, it is clear that specific experiments are definitely required to better clarify the role of these genes in our present CRC model.

## Conclusion

Our data support the hypothesis that the presently investigated *KRAS *mutations elicit, in the host *BRAF *mutated cells under study, biological consequences which may help explaining previous observations in CRC and contribute to identify novel pathways and biomarkers of potential clinical relevance.

## Methods

### Generation of stable Colo741 cell clones expressing WT, G12V and G12D KRAS mutants

The procedures for the generation of Colo741 cell clones stably transfected with construct coding for the KRAS WT and mutant isoforms, as well as the experiments needed to assess their expression and the activation of the KRAS pathway were performed according to standard protocols (see Additional Files 3, 4, 5 and 6).

### RNA extraction and quality analysis

Total RNA was isolated using RNeasy^® ^MinElute columns (Qiagen). RNA concentration and purity were determined from measuring absorbance at 260 and 280 nm; 2 μg total RNA was run on a 1% denaturing gel and 100 ng were loaded on the 2100 Bioanalyzer (Agilent, Palo Alto, CA) to verify RNA integrity.

### Amplification of RNA and array hybridization

According to the recommendations of the manufacturer, 100 ng of total RNA was used in the first-round synthesis of double-stranded cDNA. The RNA was reverse transcribed using a WT cDNA synthesis and amplification kit (Affymetrix UK Ltd., High Wycombe, UK). The resulting biotin-labeled cRNA was purified using an IVT clean-up kit (Affymetrix) and quantified using a UV spectrophotometer (A260/280; Beckman, Palo Alto, CA). An aliquot (15 μg) of cRNA was fragmented by heat and ion-mediated hydrolysis at 94°C for 35 minutes. Fragmented cRNA, run on the Bioanalyzer (Agilent Technologies, Santa Clara, CA) to verify the correct electropherogram, was hybridized in a hybridization oven (16 hours, 45°C) to a Human Gene 1.0 ST array (Affymetrix) representing whole-transcript coverage. Each one of the 28,869 genes is represented on the array by approximately 26 probes spread across the full length of the gene, providing a more complete and more accurate picture of gene expression than the 3' based expression array design. The washing and staining procedures of the arrays with phycoerythrin-conjugated streptavidin (Invitrogen) was completed in the Fluidics Station 450 (Affymetrix). The arrays were subsequently scanned using a confocal laser GeneChip Scanner 3000 7G and the GeneChip Command Console (Affymetrix).

### GeneChip microarray analysis and data normalization

Affymetrix raw data files [cell intensity (CEL) files] were used as input files in expression console environment (Affymetrix). Briefly, CEL files were processed using the Robust Multi-Array Analysis (RMA) procedure [81], an algorithm that is publicly available at . The RMA method was used to convert the intensities from the multiple probes of a probe set into a single expression value with greater precision and reduced background noise (relying on the perfect match probes only and thus ignoring the mismatch probes) and then to normalize by sketch quantile normalization. Quality assessments were also performed in the expression console environment. This procedure, based on various metrics, allowed us to identify a chip as an outlier (see for details Quality assessment of exon and gene arrays . Significance Analysis of Microarrays (SAM), Principal Component Analysis (PCA) of variance and Hierarchical Clustering (HCL), after mean scaling and log2 transformation, were performed with the software tool of The Institute for Genomic Research (TIGR) MeV [82].

Individual genes with different expression levels, among the three groups, were identified using SAM [83]. The false discovery rate expressed as q-value was used to evaluate statistical significance, and its threshold was set at 0.02 (2%). For comparison purposes, an arbitrary filter was applied excluding all genes that did not exhibit a difference in expression of at least 2-fold. Genes differentially expressed were investigated using 1) a multiclass analysis to test differences among the three groups of cells and 2) a two-class analysis within each pair groups to specify expression changes.

We used PCA to reduce the complexity of high-dimensional data and to simplify the task of identifying patterns and sources of variability in these large data sets.

The results from SAM were visualized using HCL [84].

All the microarray information has been submitted to the National Center for Biotechnology Information Gene Expression Omnibus web site .

### Pathways identification by Expression Analysis Systemic Explore (EASE)

Gene lists from Affymetrix results were examined using the EASE program, accessible via . EASE is a customized stand-alone software application with statistical functions for discovering biological themes within gene lists. This software assigns genes of interest into functional categories based on the Gene Ontology database (GO, ) and uses the Fisher's exact test statistics to determine the probability of observing the number of genes within a list of interest versus the number of genes in each category on the array. A more detailed analysis of the genes' association with physiological pathways was performed using the Kyoto Encyclopedia of Genes and Genomes (KEGG, ). Each identified process was confirmed through PubMed/Medline.

### RT-PCR analysis

Starting from about 1 μg of total RNA, cDNA was synthesized by using an Oligo(dT)20, random hexamers mix and a Superscript III first-strand synthesis system supermix for RT-PCR (Invitrogen). cDNAs were diluted 5 – 20 times, then subjected to PCR analysis.

Relative quantification was performed by real-time quantitative RT-PCR (qPCR) sybrgreen using the ABI Prism 7700 Sequence Detector (Applied Biosystems, Foster City, CA) following manufacturer's instructions. The housekeeping gene ribosomal protein L19 (*RPL19*) was used as the endogenous control for normalization because, in the microarray data, it showed in all conditions the steadiest expression when normally used housekeeping genes were compared.

To avoid possible signal production from potential contaminating genomic DNA, specific primers for each gene were designed across a common exon-exon splice junction by the Primer Express software (Applied Biosystems) (see Additional File 7). Dissociation curve analysis defined the specificity of the products by the presence of a single dissociation peak on the thermal melting curve.

## Competing interests

The authors declare that they have no competing interests.

## Authors' contributions

MMo designed research, performed research, analyzed data, wrote paper; EB, MMa, AD FR and CTS performed research; WG designed research, analyzed data, wrote paper; PC designed research, performed research, analyzed data, wrote paper.

## Supplementary Material

Additional file 1 Table S1. Probe sets significantly regulated by KRAS^G12V ^and KRAS^G12D ^Vs KRAS^G12WT ^in Colo741 cell clones. Probe sets ID, with relative gene symbols or Ensembl Transcript ID, significantly regulated by *KRAS*^*G*12*V *^or *KRAS*^*G*12*D *^in Colo741 cell clones, as determined by using SAM software with multi-class analysis. Q-values were calculated using the two-class, unpaired, option with the additional requirement of at least a 2-fold change in gene expression, relatively to *KRAS*^*WT*^-expressing clones. Click here for file

Additional file 2 Table S2. Probe sets ID significantly regulated in KRAS^G12V ^Vs KRAS^G12D ^transfected Colo741 cell clones. Probe sets ID, with relative gene symbols or Ensembl Transcript ID, significantly regulated in the comparison between the clones bearing the KRAS mutations (G12V and G12D). Q-values were calculated as in table S1. Click here for file

Additional file 3 Supplemental Methods. Methods for; generation of KRAS expression constructs, cells and cell culture, immunolocalization, establishment and selection of stable cell clones and Western blot analysis. Click here for file

Additional file 4 Figure S1. Expression and localization of recombinant KRAS proteins. *In vitro *transcription/translation of the empty vector (lane 1), *KRAS*^*WT *^(lane 2), *KRAS*^*G*12*V*^(lane 3) and *KRAS*^*G*12*D *^(lane 4), recombinant chimera RED2:*KRAS*^*WT *^(lane 5) and luciferase (control) expressing plasmids (lane 6) (A). Arrow heads point to the position of molecular mass standards whose sizes are expressed in kDa (A). Cells after 48 hours from transfection with CFP-*KRAS*^*G*12*V *^construct (B-G). Immunolocalization by an anti-pan-Ras antibody (F, G) and an Alexa514-conjugated secondary antibody giving a green signal (C, D, F, G,), direct visualization of the cyan signal pseudocolored in red (B, D, E, G) and direct visualization of the signal displayed by the nuclear dye 7-Amino Actinomycin D pseudocolored in blue (D, G). The merged fluorescence signals are shown in (D, G). Magnification of the area in the white rectangles are shown in the lower left insets of (E-G). Arrow heads point to some of the cell membrane regions displaying colocalization of the pan-RAS and CFP-*KRAS*^*G*12*V *^signals. Gamma adjustment was applied to each panel to adapt color rendering in the CMYK process. Scale bar = 50 mm. Click here for file

Additional file 5 Figure S2. Screening by semiquantitative RT-PCR analysis of Colo741 cell clones transfected with constructs expressing KRAS^WT ^(WT), KRAS^G12D ^(G12D) and KRAS^G12V ^(G12V) mRNA isoforms. For each construct both cell clones expressing the transgene (1D8-3C7-4D3) and cell clones not expressing the transgene (2G7-1H2-1B3) are shown. The latter were discarded from further experiments. For all the assayed samples, a reverse transcription PCR assays, performed omitting the reverse transcriptase, was also carried out to exclude from further analysis clones yelding a *KRAS *amplicon resulting from a plasmid integrated in the genomic DNA (false positive samples). In this example no amplicons were detected in minus reverse transcriptase assays. Primers for *GAPDH *were used to normalize the results. Click here for file

Additional file 6 Figure S3. Western blotting analysis of selected Colo741 KRAS-expressing clones. Vector-transfected clones (lane 1), *KRAS*^*WT *^(lane 2), *KRAS*^*G*12*V *^(lane 3), *KRAS*^*G*12*D *^(lane 4) and senescent human bone marrow stromal cells (lane 5) were subjected to immunoblotting to establish the global Ras expression (pan-Ras) (A) and the phosphorylation status of AKT (B) and ERK1/2 (C). Arrow points to the pAKT protein. Click here for file

Additional file 7 Table S3. Sequences accession numbers and primers. Click here for file
