# Supplementary material for: Gene expression deregulation by KRAS G12D and G12V in a BRAF V600E context
Source: Mol Cancer. 2008 Dec 16;7:92. doi: 10.1186/1476-4598-7-92 (PMC2615043; doi:10.1186/1476-4598-7-92)
Supplement: Additional file 1 — Table S1. Probe sets significantly regulated by KRASG12V and KRASG12D Vs KRASG12WT in Colo741 cell clones. Probe sets ID, with relative gene symbols or Ensembl Transcript ID, significantly regulated by KRASG12V or KRASG12D in Colo741 cell clones, as determined by using SAM software with multi-class analysis. Q-values were calculated using the two-class, unpaired, option with the additional requirement of at least a 2-fold change in gene expression, relatively to KRASWT-expressing clones. [file 1476-4598-7-92-S1.doc]

| **Probe Set ID** | **Gene Symbol / Ensembl Transcript ID** | ***KRAS^G12V^* Vs *KRAS^G12WT^*** | ***KRAS^G12D^***  **Vs *KRAS^G12WT^*** | **q-value (%)** |
| --- | --- | --- | --- | --- |
| 7897801 | *ENST00000362477* | -2,12 | *n.r.* | 0,744 |
| 7898375 | *ENST00000384659* | *n.r.* | -2,12 | 0,828 |
| 7898411 | *ENST00000384278* | *n.r.* | -2,12 | 0,828 |
| 7899436 | *SESN2* | 4,03 | 2,64 | 0 |
| 7901102 | *MMACHC* | -2,24 | *n.r.* | 0 |
| 7901867 | *USP1* | *n.r.* | 2,05 | 0,681 |
| 7902227 | *GADD45A* | 2,20 | *n.r.* | 0 |
| 7902290 | *CTH* | 2,34 | *n.r.* | 0,600 |
| 7904726 | *TXNIP* | 2,81 | *n.r.* | 0 |
| 7905581 | *S100A1* | *n.r.* | -2,07 | 0,939 |
| 7906878 | *DDR2* | 2,01 | *n.r.* | 0,733 |
| 7906900 | *ENST00000367920* | 2,11 | 2,16 | 0 |
| 7909271 | *IL24* | 3,05 | *n.r.* | 0 |
| 7911335 | *ENST00000386606* | -2,05 | *n.r.* | 0,660 |
| 7911337 | *ENST00000387392 / ENST00000386814 / ENST00000386609* | 2,35 | *n.r.* | 0 |
| 7911339 | *ENST00000387400 / ENST00000386611* | 3,03 | *n.r.* | 0 |
| 7911341 | *ENST00000387409 / ENST00000386614* | 2,47 | *n.r.* | 0,828 |
| 7912800 | *ENST00000383925* | *n.r.* | -2,12 | 0,828 |
| 7912850 | *ENST00000384782* | *n.r.* | -2,12 | 0,828 |
| 7913655 | *ID3* | *n.r.* | -2,29 | 0,681 |
| 7916432 | *DHCR24* | -2,08 | *n.r.* | 0,828 |
| 7917255 | *SSX2IP* | *n.r.* | 2,13 | 0,742 |
| 7917645 | *LOC100008589* | -2,13 | *n.r.* | 0,903 |
| 7918467 | *C1orf103* | 2,02 | *n.r.* | 0,962 |
| 7919269 | *ENST00000384687* | *n.r.* | -2,13 | 0,612 |
| 7919305 | *PRKAB2* | 2,47 | *n.r.* | 0 |
| 7919349 | *ENST00000383858* | *n.r.* | -2,13 | 0,612 |
| 7919556 | *ENST00000384476* | *n.r.* | -2,24 | 0,926 |
| 7919560 | *ENST00000384241* | -2,10 | -2,32 | 0,774 |
| 7919576 | *ENST00000384010* | *n.r.* | -2,12 | 0,828 |
| 7919584 | *HIST2H3PS2 / HIST2H2BA / HIST2H2BF* | 2,14 | *n.r.* | 0,762 |
| 7920877 | *ARHGEF2* | 2,26 | *n.r.* | 0 |
| 7924069 | *ENST00000364102* | -2,66 | *n.r.* | 0,744 |
| 7924773 | *CDC42BPA* | 2,16 | *n.r.* | 0,756 |
| 7924817 | *hypothetical protein PRO2012* | *n.r.* | 2,27 | 0,939 |
| 7924888 | *HIST3H2A* | 2,14 | *n.r.* | 0 |
| 7927631 | *DKK1* | 2,21 | *n.r.* | 0,628 |
| 7928308 | *DDIT4* | 2,71 | *n.r.* | 0 |
| 7929065 | *IFIT1* | 2,57 | *n.r.* | 0 |
| 7942779 | *ENST00000384089* | 3,16 | *n.r.* | 1,738 |
| 7943373 | *ENST00000375797* | 2,34 | *n.r.* | 0,582 |
| 7943998 | *NNMT* | 2,10 | *n.r.* | 0,681 |
| 7945864 | *ZNF195* | *n.r.* | 2,01 | 0,804 |
| 7946067 | *OR51B4* | *n.r.* | 2,28 | 0,828 |
| 7947221 | *LIN7C* | *n.r.* | 2,04 | 0,841 |
| 7947421 | *ENST00000384081* | *n.r.* | -2,62 | 1,624 |
| 7947531 | *COMMD9* | -2,25 | *n.r.* | 0,800 |
| 7949454 | *MAP3K11* | *n.r.* | -2,00 | 0,828 |
| 7951662 | *CRYAB* | *n.r.* | -2,28 | 0,717 |
| 7952205 | *MCAM* | *n.r.* | -2,42 | 0 |
| 7953218 | *RAD51AP1* | *n.r.* | 2,38 | 0,939 |
| 7953532 | *ENO2* | -3,29 | -3,20 | 0 |
| 7953873 | *OVOS2* | *n.r.* | 2,43 | 0 |
| 7954382 | *PYROXD1* | 2,48 | 2,89 | 0,768 |
| 7956120 | *ERBB3* | 2,01 | *n.r.* | 0,960 |
| 7957530 | *RL41* | 2,02 | *n.r.* | 0,717 |
| 7958913 | *OAS2* | 2,94 | *n.r.* | 0 |
| 7960359 | *RPS27* | *n.r.* | -2,24 | 0,744 |
| 7961026 | *LOC731158* | *n.r.* | 2,51 | 0 |
| 7962537 | *SLC38A2* | 2,59 | 2,26 | 0 |
| 7963054 | *TUBA1A* | -2,02 | *n.r.* | 0,773 |
| 7963575 | *ENST00000328474* | 3,01 | *n.r.* | 0 |
| 7964460 | *DDIT3* | 2,30 | *n.r.* | 0 |
| 7965956 | *NFYB* | 2,00 | *n.r.* | 0,669 |
| 7968015 | *TNFRSF19* | 2,36 | *n.r.* | 0 |
| 7970392 | *ENST00000343741* | 4,54 | *n.r.* | 0 |
| 7971422 | *ZC3H13* | 2,01 | *n.r.* | 0,828 |
| 7973530 | *PCK2* | 2,28 | *n.r.* | 0,728 |
| 7973871 | *ENST00000387392 / ENST00000386814 / ENST00000386609* | 2,35 | *n.r.* | 0 |
| 7973896 | *ENST00000383869* | *n.r.* | -2,20 | 0,711 |
| 7976073 | *FLRT2* | 2,08 | *n.r.* | 0,624 |
| 7978568 | *ENST00000383869* | *n.r.* | -2,20 | 0,711 |
| 7978905 | *ENST00000383937* | -2,77 | *n.r.* | 0,875 |
| 7979862 | *ENST00000384041* | *n.r.* | 2,63 | 0,947 |
| 7981976 | *SNORD116-14* | *n.r.* | -2,09 | 0,951 |
| 7986350 | *ARRDC4* | 2,08 | *n.r.* | 0 |
| 7986838 | *OCA2* | -2,30 | *n.r.* | 0 |
| 7987068 | *TRPM1* | -2,52 | -2,10 | 0,812 |
| 7987361 | *ZNF770* | 2,11 | *n.r.* | 0,773 |
| 7989037 | *CCPG1* | 2,38 | *n.r.* | 0,723 |
| 7994265 | *ENST00000363059* | *n.r.* | 2,16 | 0,717 |
| 7996761 | *LYPLA3* | -2,05 | *n.r.* | 0,721 |
| 7997582 | *WFDC1* | 4,62 | *n.r.* | 0 |
| 8001981 | *FHOD1* | *n.r.* | -2,07 | 1,386 |
| 8003667 | *SERPINF1* | *n.r.* | -2,26 | 0 |
| 8004167 | *FAM64A* | -2,24 | *n.r.* | 0,681 |
| 8004247 | *RNASEK* | -2,02 | *n.r.* | 0,582 |
| 8004431 | *POLR2A* | -2,16 | *n.r.* | 0,653 |
| 8004506 | *SNORA48* | -2,03 | *n.r.* | 0,768 |
| 8004521 | *MPDU1* | -2,30 | -2,04 | 0 |
| 8005547 | *ENST00000363359* | *n.r.* | -2,26 | 0,628 |
| 8005553 | *ENST00000365494* | *n.r.* | -2,26 | 0,628 |
| 8005733 | *C20orf191* | 2,01 | *n.r.* | 0,918 |
| 8010184 | *SEPT9* | 2,23 | *n.r.* | 0,900 |
| 8012856 | *ELAC2* | -2,28 | *n.r.* | 0,744 |
| 8013323 | *ENST00000364880* | *n.r.* | -2,26 | 0,628 |
| 8013325 | *ENST00000362793* | *n.r.* | -2,26 | 0,628 |
| 8013329 | *SNORD3B-2* | *n.r.* | -2,26 | 0,628 |
| 8013660 | *ALDOC* | -2,24 | *n.r.* | 0,744 |
| 8013987 | *ENST00000384284* | -2,63 | -3,42 | 0,958 |
| 8014755 | *SNORA21* | -2,07 | *n.r.* | 0,856 |
| 8017096 | *ENST00000388159* | -2,18 | -2,00 | 0,860 |
| 8019486 | *SECTM1* | *n.r.* | -2,23 | 0,693 |
| 8019631 | *ENST00000384166* | -2,95 | -2,24 | 0 |
| 8019633 | *RNU2* | -2,95 | -2,24 | 0 |
| 8019635 | *ENST00000384517* | -2,95 | -2,24 | 0 |
| 8019637 | *RNU2* | -2,95 | -2,24 | 0 |
| 8019639 | *ENST00000384692* | -2,95 | -2,24 | 0 |
| 8019641 | *ENST00000384623* | -2,95 | -2,24 | 0 |
| 8019703 | *ENST00000384374* | -2,95 | -2,24 | 0 |
| 8019705 | *ENST00000384495* | -2,95 | -2,24 | 0 |
| 8019707 | *ENST00000384758* | -2,95 | -2,24 | 0 |
| 8019709 | *ENST00000384083* | -3,02 | -2,41 | 0 |
| 8019802 | *ENST00000384642* | -3,21 | -2,24 | 0 |
| 8019877 | *SMCHD1* | 2,13 | *n.r.* | 0,756 |
| 8019885 | *SMCHD1* | 2,12 | 2,11 | 0,681 |
| 8021470 | *PMAIP1* | 2,37 | *n.r.* | 0 |
| 8021653 | *SERPINB8* | 2,31 | *n.r.* | 0,797 |
| 8022009 | *METTL4* | 2,10 | 2,06 | 0,660 |
| 8026971 | *IFI30* | *n.r.* | -2,57 | 0,951 |
| 8029530 | *APOE* | 2,04 | *n.r.* | 0 |
| 8029950 | *EHD2* | *n.r.* | -2,11 | 0,582 |
| 8030128 | *PPP1R15A* | 3,17 | *n.r.* | 0 |
| 8031514 | *ENST00000376313* | *n.r.* | -2,24 | 0,912 |
| 8035304 | *BST2* | 4,01 | *n.r.* | 0 |
| 8035865 | *ENST00000355833* | 2,23 | *n.r.* | 0,798 |
| 8040338 | *ENST00000387987* | -2,06 | *n.r.* | 1,526 |
| 8042310 | *SLC1A4* | 2,48 | *n.r.* | 0 |
| 8045279 | *ENST00000385625* | -2,02 | *n.r.* | 0,860 |
| 8045804 | *ENST00000385501* | 2,55 | *n.r.* | 0,860 |
| 8047097 | *GLS* | 2,07 | *n.r.* | 0,744 |
| 8050240 | *ODC1* | 2,04 | *n.r.* | 0,660 |
| 8053417 | *CAPG* | 4,52 | *n.r.* | 0 |
| 8056285 | *IFIH1* | 2,07 | *n.r.* | 0,636 |
| 8056792 | *ENST00000362996* | 2,42 | 2,46 | 0,956 |
| 8058390 | *RAPH1* | 2,17 | *n.r.* | 0,828 |
| 8059580 | *DNER* | -2,00 | *n.r.* | 0,800 |
| 8060344 | *TRIB3* | 2,82 | 2,26 | 0 |
| 8061447 | *PYGB* | 2,11 | *n.r.* | 0,768 |
| 8061564 | *ID1* | *n.r.* | -2,47 | 0,828 |
| 8064939 | *TXNDC13* | 2,07 | *n.r.* | 0,672 |
| 8065278 | *ENST00000354213* | -2,51 | *n.r.* | 0,681 |
| 8067029 | *KCNG1* | 3,05 | *n.r.* | 0 |
| 8069822 | *KRTAP19-1* | *n.r.* | -2,19 | 0,628 |
| 8070182 | *RCAN1* | 2,77 | *n.r.* | 0 |
| 8070632 | *CBS* | 2,49 | *n.r.* | 0 |
| 8072659 | *TOM1* | -2,07 | *n.r.* | 0,681 |
| 8078600 | *TCEA1* | 2,20 | *n.r.* | 0,834 |
| 8081838 | *CDGAP* | -2,07 | *n.r.* | 0,828 |
| 8083704 | *BRD7* | *n.r.* | 2,32 | 0,612 |
| 8087951 | *PBRM1* | 2,07 | *n.r.* | 0,681 |
| 8091283 | *PLOD2* | 2,02 | *n.r.* | 0,721 |
| 8091352 | *UBQLN4* | *n.r.* | -2,35 | 1,432 |
| 8091411 | *TM4SF1* | -3,86 | *n.r.* | 0 |
| 8092726 | *CLDN1* | 2,48 | *n.r.* | 0 |
| 8095680 | *IL8* | 3,59 | *n.r.* | 0 |
| 8100308 | *LOC728220* | *n.r.* | -2,14 | 0,875 |
| 8102800 | *SLC7A11* | 2,32 | 2,56 | 0 |
| 8103535 | *GK3P* | *n.r.* | 2,04 | 1,005 |
| 8108301 | *KIF20A* | -2,18 | *n.r.* | 0,828 |
| 8108697 | *PCDHB5* | *n.r.* | -3,38 | 0,828 |
| 8108716 | *PCDHB16* | *n.r.* | -2,82 | 0 |
| 8111417 | *SLC45A2* | -2,87 | -2,06 | 0 |
| 8111941 | *HMGCS1* | -2,39 | *n.r.* | 0 |
| 8113666 | *SEMA6A* | -2,23 | -2,01 | 0 |
| 8115327 | *SPARC* | -2,29 | -2,79 | 0 |
| 8116571 | *ENST00000339727* | 3,40 | *n.r.* | 0 |
| 8119898 | *VEGFA* | 2,47 | *n.r.* | 0 |
| 8120378 | *KIAA1586* | *n.r.* | 2,10 | 0,797 |
| 8123137 | *ACAT2* | -2,70 | *n.r.* | 0 |
| 8124380 | *HIST1H1A* | -2,36 | -2,18 | 0,800 |
| 8126629 | *GTPBP2* | 2,06 | *n.r.* | 0,834 |
| 8130211 | *SYNE1* | 2,08 | *n.r.* | 0,736 |
| 8133721 | *HSPB1* | *n.r.* | -2,24 | 0 |
| 8134821 | *MEPCE* | *n.r.* | -2,11 | 0,792 |
| 8135688 | *LSM8* | 2,27 | *n.r.* | 0,582 |
| 8139656 | *GRB10* | 2,35 | *n.r.* | 0 |
| 8141150 | *ASNS* | 2,52 | 2,04 | 0 |
| 8142912 | *FLJ14803* | 2,07 | *n.r.* | 0,893 |
| 8146859 | *ENST00000388546* | *n.r.* | -2,61 | 1,738 |
| 8146957 | *PI15* | *n.r.* | -2,08 | 0,762 |
| 8149685 | *LGI3* | -2,73 | -3,97 | 0 |
| 8150818 | *TCEA1* | 2,25 | *n.r.* | 0,800 |
| 8152119 | *NCALD* | -2,14 | *n.r.* | 0,676 |
| 8155849 | *ANXA1* | 2,51 | *n.r.* | 0,704 |
| 8165694 | *ENST00000387372* | -2,05 | *n.r.* | 0,660 |
| 8165696 | *ENST00000387392 / ENST00000386814 / ENST00000386609* | 2,35 | *n.r.* | 0 |
| 8165698 | *ENST00000387400 / ENST00000386611* | 3,03 | *n.r.* | 0 |
| 8165700 | *ENST00000387405 / ENST00000387409 / ENST00000386614* | 2,45 | *n.r.* | 0,960 |
| 8165911 | *TBL1X* | 2,02 | *n.r.* | 0,751 |
| 8174717 | *UPF3B* | 2,12 | 2,08 | 0,804 |
| 8178090 | *C6orf48* | 2,37 | *n.r.* | 0 |
| 8178884 | *HLA-DMA* | -2,03 | *n.r.* | 0,768 |
| 8179238 | *MICA* | -2,09 | *n.r.* | 0,612 |
| 8179326 | *C6orf48* | 2,37 | *n.r.* | 0 |
| 8180086 | *HLA-DMA* | -2,03 | *n.r.* | 0,768 |
| 8180192 | *Unmapped full-length transcript* | -2,28 | *n.r.* | 1,432 |

Abbreviation: *n.r.*, not regulated. Gene name symbols used are those approved by the Human Genome Organisation Gene Nomenclature Committee (<http://www.genenames.org/>).
