# Supplementary material for: Gene expression deregulation by KRAS G12D and G12V in a BRAF V600E context
Source: Mol Cancer. 2008 Dec 16;7:92. doi: 10.1186/1476-4598-7-92 (PMC2615043; doi:10.1186/1476-4598-7-92)
Supplement: Additional file 2 — Table S2. Probe sets ID significantly regulated in KRASG12V Vs KRASG12D transfected Colo741 cell clones. Probe sets ID, with relative gene symbols or Ensembl Transcript ID, significantly regulated in the comparison between the clones bearing the KRAS mutations (G12V and G12D). Q-values were calculated as in table S1. [file 1476-4598-7-92-S2.doc]

| **Affy_ID** | **Gene Symbol/ Ensembl Transcript ID** | ***KRAS^G12V^* Vs *KRAS^G12D^*** | **q-value (%)** |
| --- | --- | --- | --- |
| 7901102 | *MMACHC* | -2,33 | 0 |
| 7902553 | *IFI44* | 2,10 | 0,994 |
| 7904726 | *TXNIP* | 2,59 | 0 |
| 7909271 | *IL24* | 2,22 | 0 |
| 7911337 | *ENST00000387392 / ENST00000386814 / ENST00000386609* | 2,86 | 0 |
| 7911339 | *ENST00000387400 / ENST00000386611* | 2,88 | 0 |
| 7911341 | *ENST00000387409 / ENST00000386614* | 2,08 | 0,828 |
| 7914127 | *IFI6* | 2,81 | 0 |
| 7921516 | *SLAMF9* | -2,06 | 0,834 |
| 7924888 | *HIST3H2A* | 2,47 | 0 |
| 7929065 | *IFIT1* | 2,44 | 0 |
| 7931754 | *IDI1* | -2,16 | 0,744 |
| 7943373 | *ENST00000375797* | 2,15 | 0,582 |
| 7944867 | *ENST00000363408* | 2,10 | 0,921 |
| 7945371 | *IFITM3* | 2,19 | 0,744 |
| 7956120 | *ERBB3* | 2,29 | 0,960 |
| 7957530 | *RL41* | 2,26 | 0,717 |
| 7958884 | *OAS1* | 2,09 | 0,608 |
| 7958895 | *OAS3* | 2,14 | 0,681 |
| 7958913 | *OAS2* | 2,70 | 0 |
| 7963575 | *ENST00000328474* | 2,06 | 0 |
| 7965036 | *LOC390345* | 2,02 | 0,744 |
| 7970392 | *ENST00000343741* | 2,67 | 0 |
| 7973871 | *ENST00000387392 / ENST00000386814 / ENST00000386609* | 2,86 | 0 |
| 7976443 | *IFI27* | 2,22 | 0,824 |
| 7981181 | *SCARNA13 / SNHG10* | 2,02 | 1,366 |
| 7986350 | *ARRDC4* | 2,57 | 0 |
| 7994265 | *ENST00000363059* | -2,29 | 0,717 |
| 7996081 | *GPR56* | 2,38 | 0,856 |
| 7997582 | *WFDC1* | 5,16 | 0 |
| 8003667 | *SERPINF1* | 2,75 | 0 |
| 8010061 | *SPHK1* | 2,88 | 0 |
| 8010184 | *SEPT9* | 2,07 | 0,900 |
| 8029530 | *APOE* | 3,99 | 0 |
| 8035304 | *BST2* | 7,35 | 0 |
| 8035865 | *ENST00000355833* | 2,00 | 0,798 |
| 8036557 | *ENST00000378639* | 2,56 | 0,744 |
| 8040547 | *LOC646049* | -2,23 | 0,658 |
| 8042310 | *SLC1A4* | 3,37 | 0 |
| 8043441 | *ENST00000323432* | -2,36 | 0,660 |
| 8045279 | *ENST00000385625* | -2,16 | 0,860 |
| 8045804 | *ENST00000385501* | 3,31 | 0,860 |
| 8053417 | *CAPG* | 3,64 | 0 |
| 8069822 | *KRTAP19-1* | 2,53 | 0,628 |
| 8071036 | *S100B* | 2,13 | 0,658 |
| 8073548 | *SEPT3* | 2,99 | 0,790 |
| 8091241 | *ENST00000386072* | 2,06 | 0,956 |
| 8091411 | *TM4SF1* | -2,48 | 0 |
| 8095680 | *IL8* | 2,48 | 0 |
| 8098195 | *SC4MOL* | -3,51 | 0 |
| 8106280 | *HMGCR* | -2,37 | 0,660 |
| 8107671 | *ENST00000386243* | 2,67 | 0 |
| 8109424 | *ENST00000364735* | -2,43 | 0,704 |
| 8111941 | *HMGCS1* | -4,69 | 0 |
| 8118100 | *MICA* | -2,01 | 0 |
| 8123137 | *ACAT2* | -3,44 | 0 |
| 8130993 | *FAM20C* | 2,27 | 0,744 |
| 8133721 | *HSPB1* | 2,26 | 0 |
| 8137900 | *ENST00000382443* | 2,01 | 0,636 |
| 8137903 | *ENST00000382442* | 2,01 | 0,636 |
| 8140955 | *CDK6* | 2,03 | 0,672 |
| 8141664 | *VGF* | 2,65 | 0 |
| 8144669 | *FDFT1* | -2,01 | 0 |
| 8156321 | *SYK* | -2,68 | 0 |
| 8165696 | *ENST00000387392 / ENST00000386814 / ENST00000386609* | 2,86 | 0 |
| 8165698 | *ENST00000387400 / ENST00000386611* | 2,88 | 0 |
| 8165700 | *ENST00000387405 / ENST00000387409 / ENST00000386614* | 2,11 | 0,960 |
| 8165947 | *WWC3* | 2,75 | 0 |
| 8179238 | *MICA* | -2,12 | 0,612 |

Gene name symbols used are those approved by the Human Genome Organisation Gene Nomenclature Committee (<http://www.genenames.org/>).
