# Supplementary material for: Gene expression deregulation by KRAS G12D and G12V in a BRAF V600E context
Source: Mol Cancer. 2008 Dec 16;7:92. doi: 10.1186/1476-4598-7-92 (PMC2615043; doi:10.1186/1476-4598-7-92)
Supplement: Additional file 3 — Supplemental Methods. Methods for; generation of KRAS expression constructs, cells and cell culture, immunolocalization, establishment and selection of stable cell clones and Western blot analysis. [file 1476-4598-7-92-S3.doc]

#### Generation of KRAS expression constructs.

The pcDNA3-*KRAS^G12V^* was a kind gift from Dr. Natalia Ignatenko, University of Arizona. This construct contains the *KRAS* cds with the GGT/GTT transversion at the codon 12 and its insert was used as template to generate, by polymerase chain reaction, the complete cds sequences for *KRAS^WT^* (wild-type), *KRAS^G12V^* and *KRAS^G12D^* (GGT/GAT transition). These cds were cloned in the pcDNA3.1 plasmid (Invitrogen, San Diego, CA) with the use of BamHI/HindIII restriction enzymes, whose recognition sites were introduced by synthetic PCR primers. The DNA sequences were verified by automated sequencing using a 377 Perkin Elmer sequencer. All sequencing reactions were performed by using the BigDye terminator cycle sequencing kit (Perkin Elmer, Waltham, MA). In order to verify the ability of these constructs to generate *KRAS* proteins of the expected size an *in vitro* transcription and translation experiment was performed in the presence of [^35^S]methionine and [^35^S]cysteine (GE Healthcare, Uppsala, Sweden) according to the manufacturer's protocol by using the TNT Quick-Coupled transcription-translation system (Promega Corporation, Madison, WI). The radioactive-labeled proteins were analyzed by SDS gel electrophoresis and showed a molecular mass in agreement with the expected 21 kDa for the KRAS proteins and with the sum of 21 kDa of KRAS plus the 25.8 kDa of the DsRed2 protein, about 47 kDa, expected for the chimera *KRAS*:RED2 after cloning of the *KRAS* cds in the pDsRed2-N1 vector (Clontech, Mountain View, CA) (Figure S1A). Digital images were acquired using the Cyclone phosphor imaging system (Perkin Elmer). With the sole purpose to verify the correct subcellular localization of the KRAS proteins, the plasmids pcDNA3.1-RFP:*KRAS*, pcDNA3.1-CFP:*KRAS* and pcDNA3.1-EYFP:*KRAS* were generated by subcloning, respectively, the HcRed (red fluorescent protein), the CFP (cyan fluorescent protein) and EYFP (enhanced yellow fluorescent protein) cds in frame at the 5’ end of the *KRAS^WT^*, *KRAS^G12V^* and *KRAS^G12D^* cds of the pcDNA3.1 vector. These constructs were not used to generate the stable transfected cell clones used for the expression array experiments.

#### Cells and cell culture methods.

The human colorectal carcinoma cell line Colo741 was obtained from ATCC. Cells were cultured in a growth medium RPMI1640 (Euroclone, Milano, Italy) supplemented with 10% fetal calf serum (Invitrogen), 1% glutamine, and 1% penicillin-streptomycin. Cultures were incubated at 37°C in a 5% CO_2_ atmosphere. The *KRAS, APC* mutation and microsatellite status of these cells were determined by Suter and coworkers (29) (see also [www.sanger.ac.uk/genetics/CGP/cosmic/](http://www.sanger.ac.uk/genetics/CGP/cosmic/)). We determined that Colo741 cell line has an effective doubling time of 37.5 hours and that is aneuploid as assessed by flow cytometry and multicolor fluorescent in situ hybridization (FISH) (data not shown).

#### Immunolocalization.

Cells were fixed in 3% paraformaldehyde in phosphate buffered saline (Na_2_HPO_4_ 8.1 mM, NaCl 136,89 mM, KH_2_PO_4_ 1,47 mM, KCl 2.68 mM), pH 7.6 containing 2% sucrose for 5 min at room temperature. After rinsing in phosphate buffered saline, cells were permeabilized in a solution containing 20 mM Hepes pH 7.4, 300 mM sucrose, 50 mM NaCl, 3 mM MgCl2 and 0.5% Triton X-100 for 5 min at 0°C. Non-specific binding was prevented by incubation with pure Goat serum for 30 min at 0°C. Non-specific binding was prevented by incubation with pure Goat serum for 30 min at 0°C. Slides were incubated with the anti-PanRAS (RAS10) monoclonal antibody (Millipore, Billerica, MA), in phosphate buffered saline supplemented with 10% goat serum (antibody dilution buffer) for 2 hours at 0°C. After extensive rinsing in phosphate buffered saline, slides were incubated for 30 min with FITC-conjugated goat anti-mouse antibody in antibody dilution buffer. Nuclei were stained with 7-Amino Actinomycin D (Invitrogen) 10 mg/ml in PBS for 20 min at 37°C. Routine observations and image acquisition were performed with an Axiovert 200M microscope using the Axiovision Software (Carl Zeiss, Jena, Germany). A representative image displaying a partial overlap of the fluorescent signal generated from the chimeric CFP:*KRAS^WT^* chimera with the signal generated from the panRAS antibody is shown (Figure S1F, G). Negative control experiments performed omitting the panRAS antibody do not show any signal (Figure S1D, E).

#### Establishment and selection of stable cell clones.

Transfection of the pcDNA3.1-*KRAS^WT^*, *KRAS^G12V^* and *KRAS^G12D^* constructs into Colo741 cells was performed with *Fugene* (Roche, Basel, Switzerland) according to the manufacturer's instructions. Fourthy-eight hours after transfection, the cells were selected in medium containing 200 µg/ml G418 (Invitrogen) for 4 weeks and then cloned using limiting dilution. After about additional 4 weeks from the transfection, the selected clones were screened by PCR using a forward primer annealing in the *KRAS* cds and a reverse primer annealing in the Bovine growth hormone (BGH) reverse priming site of the cloning vector (Figure S2). Possible contamination by genomic DNA was verified by an "RT minus" control, consisting of a mock cDNA synthesis reaction performed omitting the reverse transcriptase enzyme (Figure S2). The transcript of the housekeeping gene *GAPDH* was used as the endogenous control for normalization. Primers and cDNA sequence accession numbers for each assayed gene are listed in Table S3 (see Additional File 7).

#### Western blot analysis.

The Pierce BCA protein assay (Thermo Scientific, Rockford, IL) was used to estimate total cell protein concentration from freshly isolated cell lysates. Equal amounts of total protein were separated by SDS-polyacrylamide gel electrophoresis, transferred onto nitrocellulose paper by electroblotting, subsequently immunoblotted using one of the following antibodies: the monoclonal anti-PanRAS (RAS10) (Millipore); the polyclonal Phospho-p44/42 (Tyr202/Tyr204) Map Kinase (Cell signaling technology, Danvers, MA); the polyclonal Phosphoplus Akt (S73) (Cell signaling technology). Antigen-antibody complex were revealed using horseradish peroxidase (HRP)-conjugated secondary antibody (GE Healthcare) and the enhanced chemiluminescence system (GE Healthcare). Membranes were then exposed to ECL hyperfilm (GE Healthcare) to collect light emission and document membrane images (Figure S3).
