# Supplementary figures and images for: Gene expression deregulation by KRAS G12D and G12V in a BRAF V600E context
Source: Mol Cancer. 2008 Dec 16;7:92. doi: 10.1186/1476-4598-7-92 (PMC2615043; doi:10.1186/1476-4598-7-92)

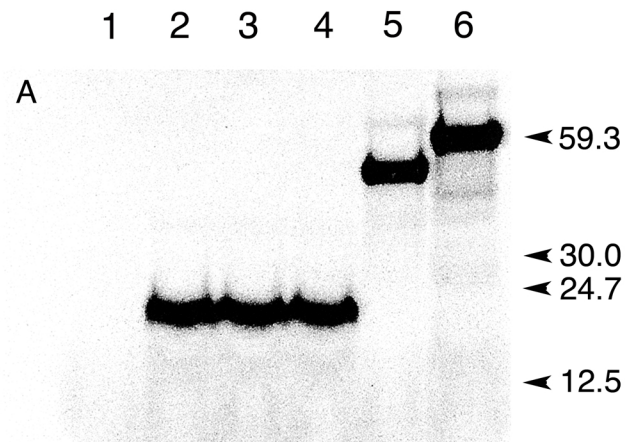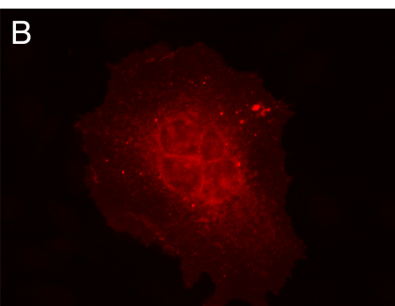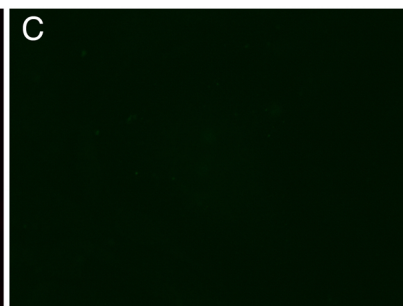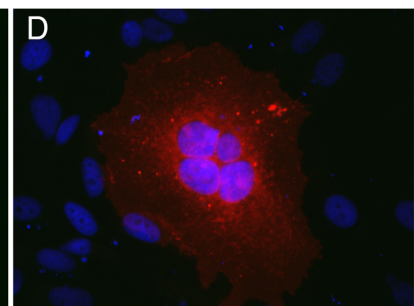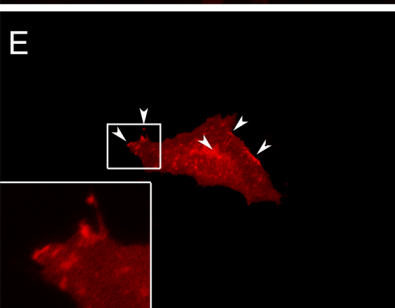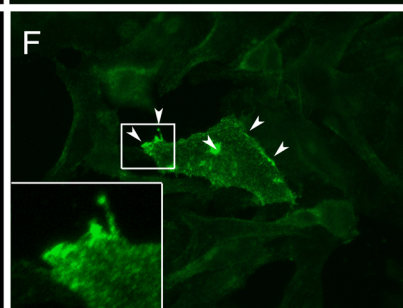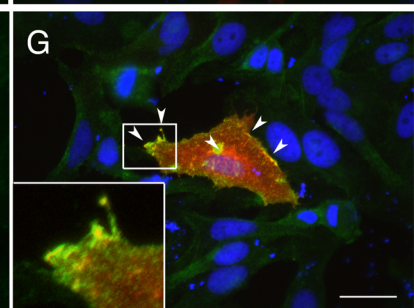

Supplement: Additional file 4 — Figure S1. Expression and localization of recombinant KRAS proteins. In vitro transcription/translation of the empty vector (lane 1), KRASWT (lane 2), KRASG12V(lane 3) and KRASG12D (lane 4), recombinant chimera RED2:KRASWT (lane 5) and luciferase (control) expressing plasmids (lane 6) (A). Arrow heads point to the position of molecular mass standards whose sizes are expressed in kDa (A). Cells after 48 hours from transfection with CFP-KRASG12V construct (B-G). Immunolocalization by an anti-pan-Ras antibody (F, G) and an Alexa514-conjugated secondary antibody giving a green signal (C, D, F, G,), direct visualization of the cyan signal pseudocolored in red (B, D, E, G) and direct visualization of the signal displayed by the nuclear dye 7-Amino Actinomycin D pseudocolored in blue (D, G). The merged fluorescence signals are shown in (D, G). Magnification of the area in the white rectangles are shown in the lower left insets of (E-G). Arrow heads point to some of the cell membrane regions displaying colocalization of the pan-RAS and CFP-KRASG12V signals. Gamma adjustment was applied to each panel to adapt color rendering in the CMYK process. Scale bar = 50 mm. [file 1476-4598-7-92-S4.pdf]

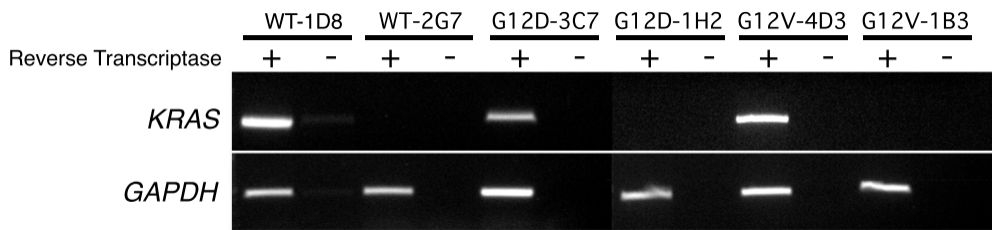

Supplement: Additional file 5 — Figure S2. Screening by semiquantitative RT-PCR analysis of Colo741 cell clones transfected with constructs expressing KRASWT (WT), KRASG12D (G12D) and KRASG12V (G12V) mRNA isoforms. For each construct both cell clones expressing the transgene (1D8-3C7-4D3) and cell clones not expressing the transgene (2G7-1H2-1B3) are shown. The latter were discarded from further experiments. For all the assayed samples, a reverse transcription PCR assays, performed omitting the reverse transcriptase, was also carried out to exclude from further analysis clones yelding a KRAS amplicon resulting from a plasmid integrated in the genomic DNA (false positive samples). In this example no amplicons were detected in minus reverse transcriptase assays. Primers for GAPDH were used to normalize the results. [file 1476-4598-7-92-S5.pdf]

1 2 3 4 5

A

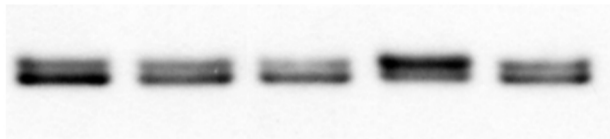

panRas

B

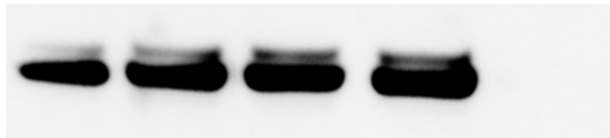

pErk1/2

C

→

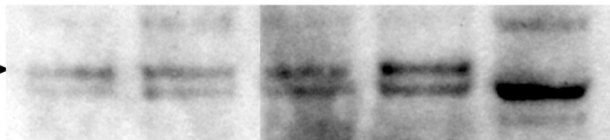

pAkt

Supplement: Additional file 6 — Figure S3. Western blotting analysis of selected Colo741 KRAS-expressing clones. Vector-transfected clones (lane 1), KRASWT (lane 2), KRASG12V (lane 3), KRASG12D (lane 4) and senescent human bone marrow stromal cells (lane 5) were subjected to immunoblotting to establish the global Ras expression (pan-Ras) (A) and the phosphorylation status of AKT (B) and ERK1/2 (C). Arrow points to the pAKT protein. [file 1476-4598-7-92-S6.pdf]
