# Supplementary material for: Gene expression deregulation by KRAS G12D and G12V in a BRAF V600E context
Source: Mol Cancer. 2008 Dec 16;7:92. doi: 10.1186/1476-4598-7-92 (PMC2615043; doi:10.1186/1476-4598-7-92)
Supplement: Additional file 7 — Table S3. Sequences accession numbers and primers. [file 1476-4598-7-92-S7.doc]

| **Gene** | **Accession number** | **Forward (5'-3')** | **Reverse (5'-3')** |
| --- | --- | --- | --- |
| **SQ-RT-PCR** |  |  |  |
| *KRAS* | NM_004985 | GAGGATTCCTACAGGAAGCAAG | TAGAAGGCACAGTCGAGG |
| *GAPDH* | M32599 | ACCACAGTCCATGCCATCAC | TCCACCACCCTGTTGCTGTA |
| **Real-Time PCR** |  |  |  |
| *BST2* | NM_004335 | GCAGTGAGATCCCAGGAAGCT | CAAGGGAATGTTCAAGCGAAA |
| *DDIT3* | NM_004083 | CAGCGACAGAGCCAAAATCA | GCCATCTCTGCAGTTGGATCA |
| *FDFT1* | NM_004462 | GGGATGGCAGAGTTTTTGGA | GACCAGCCCAGCAACATAGTG |
| *HMGCR* | NM_000859 | TATCAGCTGCACCATGCCA | TCTTTGCATGCTCCTTGAACA |
| *HMGCS1* | NM_002130 | GCACAGCTGCTGTCTTCAATG | AACTACCAGGGCATACCGTCC |
| *HSPB1* | NM_001540 | AGGATGGCGTGGTGGAGAT | AGCGTGTATTTCCGCGTGA |
| *METTL4* | NM_022840 | TCCACACAAAAAGCCCTACGA | GGGAGCACGTTTACATCTGCA |
| *OAS1* | NM_016816 | ACCTGAGAAGGCAGCTCACG | GGTCTCCACCACCCAAGTTTC |
| *PPP1R15* | NM_014330 | CGCCCAGAAACCCCTACTC | CAGACAGCCAGGAAATGGACA |
| *RAD51P1* | NM_006479 | TGCGGCCTGTGAGACATAAG | TCCTTTGGTGCTGTTCTGGAT |
| *RPL19 (82)* | NM_000981 | GTCTGGTTAGACCCCAATGAG AC | GCAAAATCCTCATTCTCCTCATC |
| *SEMA6A* | NM_020796 | CATCTTCAACAGGCCATGGTT | GCAGCTGTGTCCACTGCAAT |
| *SESN2* | NM_031459 | AATACCATCGCCATGCACAG | GCTGGTTCACCTCCCCATAA |
| *SMCHD1* | NM_015295 | AATCTGGATGCGGCCAATC | GCCCCCAAACTTTCCATTACT |
| *SYK* | NM_003177 | ACGCCAAGATCAGTGATTTCG | TCCGGAGCGTACCACTTGAC |
| *TM4SF1* | NM_014220 | GTCATTGTGGCAGCCCTTG | CGGACCATGTGGAGGTATCC |
| *TRIB3* | NM_021158 | ACTGTCACCAGCACGGTCTG | GGAATCATCTGGCCCAGTCA |
| *VEGFA* | NM_001025366 | GCCCACTGAGGAGTCCAACA | TCCTATGTGCTGGCCTTGGT |

Gene name symbols used are those approved by the Human Genome Organisation Gene Nomenclature Committee (<http://www.genenames.org/>).
